# Enhancing cognition and well-being by treating the malocclusion unilateral posterior crossbite: preliminary evidence by a single case study

**DOI:** 10.3934/Neuroscience.2025007

**Published:** 2025-04-25

**Authors:** Laura Mandolesi, Noemi Passarello, Eillyn Leiva Ramirez, Deny Menghini, Patrizia Turriziani, Teresa Vallelonga, Francesco Aristei, Angela Galeotti, Stefano Vicari, Vito Crincoli, Maria Grazia Piancino

**Affiliations:** 1 General and Experimental Psychology Laboratory, Department of Humanities, University of Naples “Federico” II, Naples, Italy; 2 Department of Surgical Sciences, C.I.R. Dental School, University of Turin, Turin, Italy; 3 Child and Adolescent Neuropsychiatry Unit, Bambino Gesù Children's Hospital, IRCCS, Rome, Italy; 4 Neuropsychology Lab, Department of Psychology, Educational Sciences and Human Movement, University of Palermo, Palermo, Italy; 5 Orthodontic Private practice, Koelliker Hospital and Nursing Home, Turin, Italy; 6 Dentistry Unit, Management Innovations, Diagnostics and Clinical Pathways, Bambino Gesù Children's Hospital, IRCCS, Rome, Italy; 7 Department of Life Sciences and Public Health, Catholic University of the Sacred Heart, Rome, Italy; 8 Department of Basic Medical Sciences, Neuroscience and Sense Organs, University of Bari, Bari, Italy

**Keywords:** mastication, spatial abilities, memory, graphic fluency, children

## Abstract

Scientific investigations have increasingly revealed the intricate connection between masticatory function and cognitive functioning, as well as psychological well-being. Addressing malocclusions, such as the common unilateral posterior crossbite, during early developmental stages, emerges as an effective strategy for gaining health. This study aims to evaluate the efficacy of treating unilateral posterior crossbite by utilizing a function generating bite (FGB) appliance in a 12-year-old child, focusing on its impact on specific cognitive domains. Through meticulous pre- and post-treatment assessments, encompassing global cognitive activity (Colored Progressive Matrices), verbal and spatial working memory (Digit Span and Corsi Block-Tapping Test), and graphic fluency (modified Five Points Test), this research aims to elucidate the cognitive benefits associated with FGB treatment. Results unveil that a mere seven-month application of FGB effectively rectified the malocclusion while concurrently yielding notable improvements in the cognitive abilities under scrutiny. Additionally, post-treatment observations revealed enhanced well-being and sleep quality, further emphasizing the multifaceted benefits of such interventions. Although this study presents findings from a singular case, it serves as a catalyst for further exploration into the intricate interplay between masticatory function and cognitive performance. Such endeavors are vital for advancing holistic healthcare practices that integrate dental care with cognitive and psychological considerations, thereby fostering comprehensive well-being and optimal development.

## 1. Introduction

Experimental and clinical studies evidence a strong relation between masticatory function and cognition [1], opening new horizons of research and intervention in the field of psychological well-being and health. Mastication has been identified as a cognitive stimulant, enhancing alertness, attention, neural processing speed, and learning and memory performance [2]–[4]. Neuroimaging studies further support the connection, demonstrating that mastication can influence blood-oxygen-level-dependent (BOLD) signals in brain regions activated during cognitive tasks [5],[6]. Although the physiological mechanisms of this link have not yet been fully explored, several experimental studies demonstrated that chewing activates the noradrenergic system by means of the locus coeruleus, thus promoting a high state of alertness and attention [6],[7]. Moreover, for the first time, Kandel and collaborators have evidenced in animals that the noradrenergic locus coeruleus releases dopamine in the dorsal hippocampus [8], providing also new insights into dopamine involved in chewing. This discovery led to the exploration of the effects of chewing on specific cognitive domains, such as attention and spatial memory, and, at the same time, provided a reasonable interpretation of all those findings that highlighted cognitive impairment associated with impaired chewing [4]. In this framework, animal studies have demonstrated a decreased number of hippocampal pyramidal neurons and reduced neurogenesis, as well as impaired memory and spatial orientation associated with impaired mastication [1],[9],[10]. In humans, scientific research has begun to investigate, on one hand, the effects of correct chewing on the brain and cognitive functioning and, on the other, the correlation between altered chewing cycles and cognitive impairment. Particular attention is paid to the pediatric age, in which orthodontic assessments and treatments, even at the early stages of life, can play a pivotal role in gaining well-being and preventing or minimizing potential cognitive impairment associated with altered chewing [1]. Among the causes for chewing pattern alterations, which can cause significant negative effects on the brain activity, there are some malocclusions, such as the common unilateral and bilateral posterior crossbite [11],[12], which are defined as the unilateral or bilateral inversion of the occlusal relationship between upper and lower posterior teeth. Previous studies evaluating children with crossbites have shown asymmetry in masticatory muscle activity, decreased muscular activation intensity of bite force, and asymmetric growth of orofacial structures [13], also highlighting how important the effects of correct chewing are on the harmony of the entire body. Moreover, because crossbites develop during eruption of the primary dentition, they have a powerful influence on the developing central pattern generator, establishing the anomalous reverse-sequencing type of chewing pattern [12].

To date, most of the evidence demonstrating a relationship between chewing and cognition comes from experimental studies on animals. Despite this, the first clinical observations on the correlation between crossbite and alteration of cognitive abilities are beginning to emerge. However, a structured investigation with an interdisciplinary character is still lacking.

This preliminary investigation, employing a single-case design, seeks to contribute empirically to the nascent field of research examining the cognitive activity of children with unilateral posterior crossbite. Focusing on a 12-year-old subject presenting a unilateral posterior crossbite malocclusion, our principal aim is to assess the effects of correction with a function generating bite (FGB) appliance on masticatory function and specific cognitive domains. Leveraging standardized neurophysiological tests, including the Colored Progressive Matrices (CPM) [14], the Digit Span Test and the Corsi Block-Tapping Test [15], and the modified Five Points Test (m-FPT) [16], our study offers a rigorous exploration of the intricate relationship between masticatory function and cognitive outcomes within the context of unilateral posterior crossbite. We have also chosen to include assessments of overall well-being and quality of life before and after treatment, incorporating the Very Short Well-being Questionnaire (VSWQ) [17] and the Sleep Disturbance Scale for Children (SDSC) [18]. In the broader scientific landscape, this work contributes substantively to advancing our understanding of these interrelated dimensions.

## 2. Methods

### 2.1. Participant

The participant in this study was a child, 149 months old, with a seven-year educational background. The participant had a right posterior unilateral crossbite malocclusion and underwent an initial orthodontic evaluation with photographs, radiographs, cephalometrics, study models, and chewing patterns recording. Notably, the individual had no history of past or present developmental, neuropsychiatric, or psychological disorders. He was recruited as a volunteer, and written information consent was signed by his parents. The study was approved by the Local Ethics Committee of the “Federico II” University of Naples (protocol number: 11/2020) and by the Institutional Review Board of the University Hospital “Health and Science Complex Turin, Italy n. CS/246 and was carried out in accordance with the Declaration of Helsinki.

The participant's demographics pre- and post-treatment are reported in Table 1.

**Table 1.** The participant's demographics pre-treatment (baseline, A) and post-treatment (treatment, B).

	Age (months)	Years in a school environment according to the organization in Italy
Baseline (A)	149	7
Treatment (B)	156	7

### 2.2. Procedure

The procedure for our single-case study adheres to an AB structure, where A represents the baseline and B denotes the treatment phase. The treatment spanned seven months and was executed according to the details outlined in paragraph 2.2.2. Each of the two evaluation sessions (baseline and treatment) lasted 2 hours.

During the evaluations, a neuropsychological assessment was conducted aimed at analyzing general cognitive abilities through the Colored Progressive Matrices (CPM) [14] and specific cognitive abilities such as verbal and visuospatial working memory. For these two domains, the Digit Span Test and the Corsi Block-Tapping Test [15],[19] were used. Graphic fluency was assessed using the modified Five Points Test (m-FPT) [16]. Furthermore, health and well-being were measured using the Very Short Well-being Questionnaire (VSWQ) [17] and the Sleep Disturbance Scale for Children (SDSC) [18]. Chewing pattern recording and kinematic analysis were performed on each of the two sessions.

#### 2.2.1. Neuropsychological and well-being evaluation

*Colored Progressive Matrices (CPM)*. CPMs are widely used for neuropsychological assessment in children. This test is designed to assess general cognitive abilities, particularly non-verbal reasoning skills and abstract thinking. It consists of a series of matrices, each featuring a pattern with one missing element. The participant's task is to identify the missing element from a set of options, demonstrating their ability to recognize and apply logical patterns [14],[20].

*Digit Span Test (DS)*. The DS is a widely utilized neuropsychological assessment designed to evaluate an individual's working memory capacity, specifically the auditory-verbal component. Developed as part of the Wechsler Intelligence Scale, the test involves presenting a sequence of digits to the participant, who is then required to recall the sequence in the same order (Digit Span Forward) or in reverse order (Digit Span Backward). The length of the digit sequences gradually increases, challenging the participant's ability to temporarily hold and manipulate information in their working memory [15].

*Corsi Block-Tapping Test (CBT)*. The CBT is a widely used neuropsychological tool designed to assess visuospatial working memory. The test involves a series of spatial sequences, where a sequence of blocks is tapped by the examiner, and the participant is then required to reproduce the sequence by tapping the same blocks in the correct order. The difficulty increases as the length of the sequences progresses. This task evaluates an individual's ability to temporarily store and manipulate visuospatial information in their working memory, providing valuable insights into their visuospatial processing and organizational skills [15].

*Modified Five Points Test (MFPT)*. The MFPT is a neuropsychological assessment tool designed to evaluate graphic fluency and executive functions. Participants are required to connect five dots arranged in a cross-like configuration, generating as many unique designs as possible within a specified time limit. The test assesses an individual's ability to create novel and creative graphic patterns, offering insights into their executive functioning, cognitive flexibility, and problem-solving skills. It is widely used in clinical, educational, and research settings to assess cognitive abilities related to graphic fluency and creative problem-solving. The MFPT evaluates several parameters, including the number of unique designs (MFTP_Production), production of designs with lines that fail to connect dots (MFTP_Rule Breaking), and repetitions of design production with lines that fail to connect dots (MFTP_Perseverations) [21],[22].

*Very Short Well-being Questionnaire (VSWQ)*. The VSWQ is a concise yet comprehensive tool designed to measure an individual's subjective well-being and psychological health. This self-report questionnaire consists of a small number of items, focusing on key aspects of well-being such as emotional state, life satisfaction, and overall happiness. Participants respond to the items on a Likert scale, providing a quick and efficient assessment of their subjective well-being [17].

*Sleep Disturbance Scale for Children (SDSC)*. The SDSC is a widely used tool for assessing sleep disturbances and related issues in children. This parent-reported questionnaire aims to capture various aspects of sleep, including bedtime resistance, sleep duration, night waking, parasomnia, and overall sleep-related behaviors. Comprising a range of items, the SDSC provides a comprehensive overview of a child's sleep patterns and potential sleep-related difficulties. By gathering information on both quantitative and qualitative aspects of sleep, the SDSC is a valuable instrument in clinical and research settings, aiding in the identification and evaluation of sleep disturbances in children [18].

#### 2.2.2. Orthodontic evaluation

**Figure 1.**
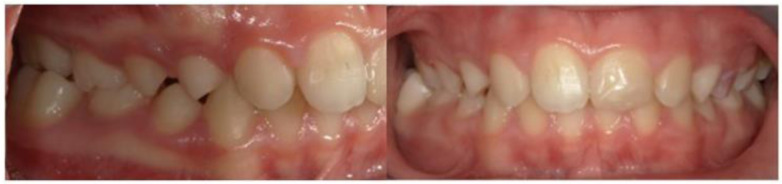
Pre-treatment image with unilateral right posterior crossbite.

*Occlusion*. The occlusal relationship was diagnosed on the study models: the patient showed a unilateral posterior right crossbite in the frontal plane involving the first right upper and lower permanent molars, a bilateral neutral relationship in the sagittal plane between the upper and lower first molars, and an increased anterior overbite (Figure 1).

The analysis of the cranial structure (cephalometry) is reported in Table 2.

**Table 2.** Participant's pre-treatment cephalometric (cranial structure) data.

**Pre-treatment DATA**		**Mean**	**Range**	**SD**
**Cephalometric analysis**	**SpP^GoGn**	28	20°+5°	8
	**SpP^F**	−3	5°–3°	−8x
	**SpP^Oc**	13	8°–3°	5
	**A:Po**	+11 mm	+2–3 mm	(+9xx)
	**ANB**	+3°	2°	(+1)

Note: SpP^GoGn, angle between superior maxilla (SpP) and the body of the mandible (GoGn) in order to evaluate the maxillary divergency, according to Schudy [23]; SpP^F, angle between superior maxilla (SpP) and condyle-or- bital plane (CoOr), SpP^Oc, angle between superior maxilla (SpP) and occlusal functional plane (Oc), in order to evaluate the orientation of the occlusal plane; AN^B, sagittal cranial relationship (the relationship in the sagittal plane between the upper maxilla and the mandible with respect to the cranial base as reference), according to Steiner [24]; A:Po, sagittal maxillary relationship (the relationship in the sagittal plane between the upper maxilla and the mandible, using the upper maxilla as reference bone), according to Schudy [23].

*Chewing pattern registration*. The recordings of the chewing cycles were carried out for the participant. The child was seated in a chair with a 90° backrest, and the environment was comfortable and quiet. Each recording began with the child in maximal intercuspation position. The child was asked to hold this position with the test bolus on the tongue, prior to starting the recording. The participant was instructed to chew a soft bolus (chewing gum) and then a hard bolus (wine gum), deliberately on the right and left sides. The duration of each test was 10 s, and each set was repeated three times. The soft bolus was a piece of chewing gum, and the hard bolus was a wine gum, both of which were the same size (20 mm in length, 1.2 mm in height, and 0.5 mm in width) but of different weights (2 g for the soft bolus and 3 g for the hard bolus). The wine gum was chosen to provide a rubber-like resistance without sticking to the teeth.

*Kinematic analysis*. Mandibular movements were measured with a kinesiograph (K7, Myotronics Inc. Tukwila, Washington, USA), which measures jaw movements within an accuracy of 0.1 mm. Multiple sensors (Hall effect) in a light-weight array (113 g) tracked the motion of a magnet attached to the midpoint of the lower incisors. The kinesiograph was interfaced with a computer for data storage and subsequent analysis. Chewing cycles were divided into non-reverse and reverse, based on the vectoral direction of closure. The closure angle was measured between a straight line obtained by a robust regression procedure on the last part of the curve (from 2.0 to 0.1 mm from the closing point in the vertical direction) and the horizontal line of the side of mastication. Next, cycles with a closure angle larger than 908 were grouped in the reverse set.

#### 2.2.3. Orthodontic evaluation

**Figure 2.**
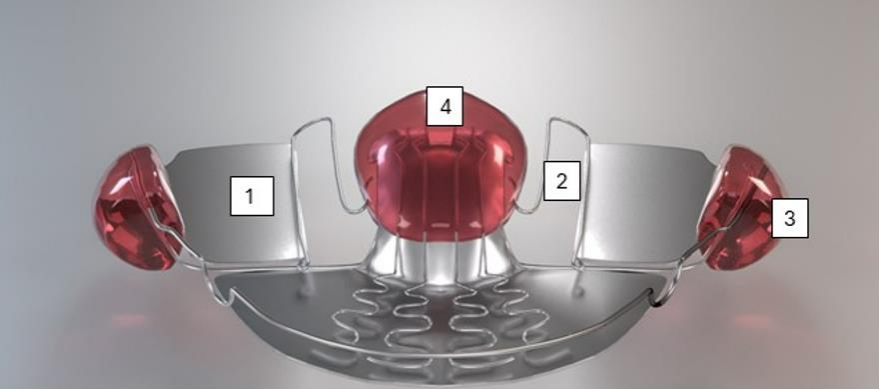
Function generation bite before activation with stainless steel resilient bite planes (1), expansion springs (2), buccal shields (3), and palatal plate (4). See the Methods section for the explanation.

The participant was treated with FGB appliances (Figure 2). The appliance was individually manufactured in acrylic resin and resilient stainless steel, with occlusal metallic bite planes preventing the teeth from intercuspal contacts, aligning the occlusal plane and allowing the mandible to self-reposition in the three planes of space. The expansion springs were designed to lie 2 mm below the equator of the posterior maxillary teeth at rest and to reach it during swallowing in order to transmit the force generated by the activated masseter muscles through the buccal shields.

The unilateral posterior crossbite was considered corrected when the buccal cusps of the upper teeth, which were previously in crossbite, overlapped the lower teeth, thus providing the appropriate physiological stimuli from peripheral receptors and proprioceptors. In the active phase of treatment, the child was instructed to wear the appliance as much as possible during the day and night, except during mealtimes. After the correction of the malocclusion, the appliance was worn at night only for retention.

During treatment, the expansion springs were activated in accordance with the need to achieve transverse growth, as well as to adapt the appliance to the changes occurring in a developing individual. The orthodontic forces exerted by the expansion springs of this appliance, which come from the patient's neuromuscular activation during swallowing, are intermittent, self-regulating, and, therefore, individual, and are suitable for the growing child.

#### 2.2.4. Data analysis

In this single-case study, the data analysis involved descriptive analyses of cognitive test scores and well-being questionnaires. The participant's performance was scrutinized in both the baseline and treatment phases. Bar graphs and charts were used to assess individual performance trends through cognitive assessments and well-being measures. Neuropsychological test scores were also evaluated by calculating the standardized (Z) score relative to normative data, using samples that were comparable to the age and education level of the participant. We also reported an Improvement index (Δ) by simply calculating the difference between treatment and baseline z-scores for each test. All statistical analyses and graphical representations were performed using the R software (v. 4.1.2).

## 3. Results

### 3.1. Comparison to normative data

Participant raw scores were transformed into z-scores, allowing for a standardized comparison to normative data. Prior to treatment, all test scores fell within the normative range, indicating that the participant's performance was generally consistent with expected levels for his age group (Table 2). However, it is noteworthy that the raw score for sleep difficulties (SDSC) at baseline (raw score = 53; normative mean = 35.05; normative SD = 7.7) exceeded the expected range.

Following treatment, all test scores remained within the normative range, but with improvements in scores (Table 3). It is important to highlight that after treatment, the participant's sleep quality scores demonstrated a notable improvement, as they now fall within the normative range (raw score = 43; normative mean = 35.05; normative SD = 7.7).

**Table 3.** Participant baseline raw scores in all tests, normative mean, and SD.

**Domain**	**Test**	**Pre-treatment raw score**	**Post-treatment raw score**	**Normative mean**	**Normative SD**
Cognitive level	CPM	32	33	30.81	2.8
Working memory	DS	5	7	4.4	1
	CBT	6	6	4	1
Graphic fluency	FPT_Production	35	49	18.84	9.07
	FPT_Rule Breaking	1	0	0.16	0.5
	FTP_Perseverations	1	0	0.8	1.2
Well-being	VSWQ	14	16	16.38	2.87
	SDSC	**53**	43	35.05	**7.7**

Note: CPM = Colored Progressive Matrices; DS = Digit Span Test; CBT = Corsi Block-Tapping Test; FPT = Five Points Test; VSWQ = Very Short Well-being Questionnaire; SDSC = Sleep Disturbance Scale for Children.

### 3.2. Cognitive level and working memory assessment

**Figure 3.**
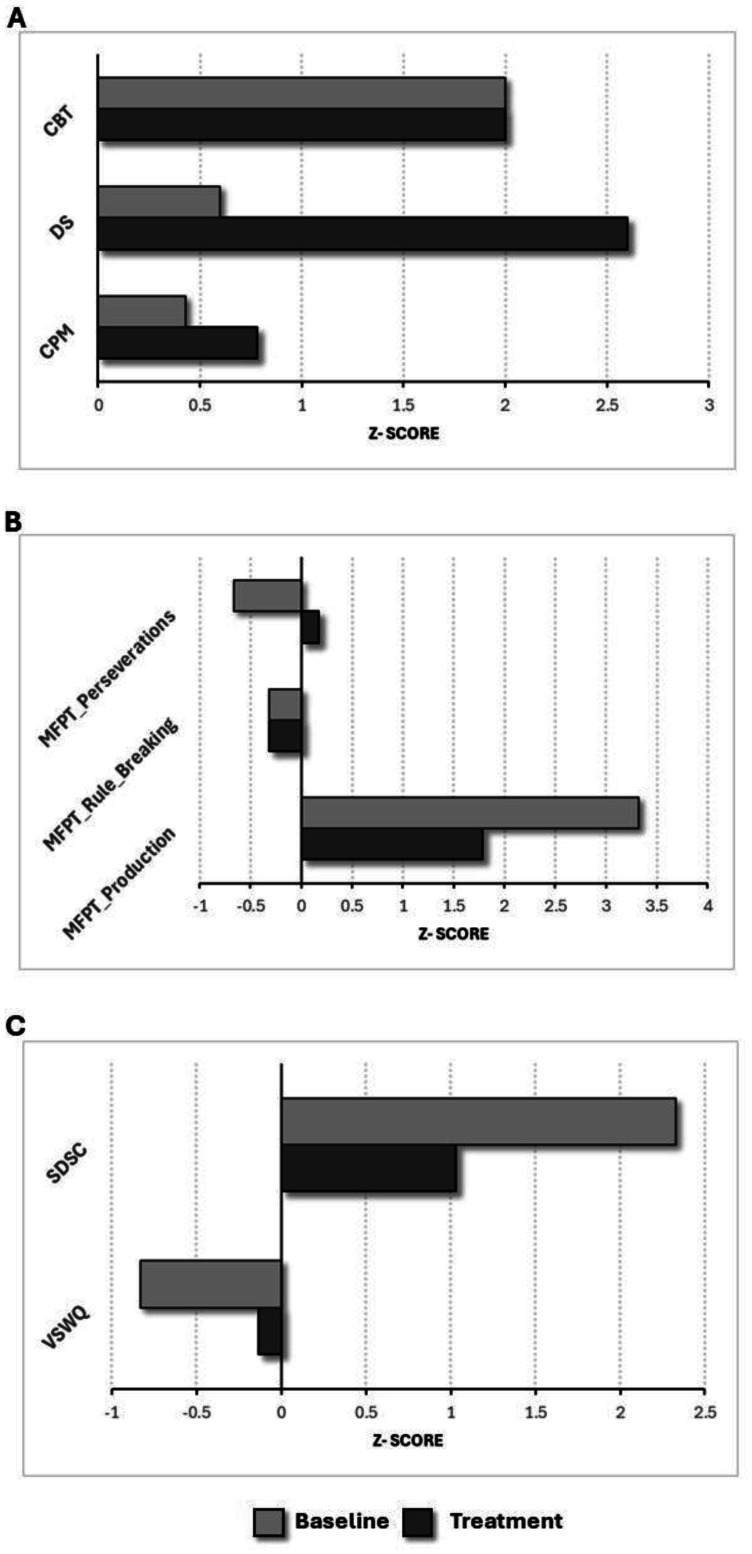
Baseline vs. treatment z-scores for general cognitive level, verbal, and visual working memory (A); graphic fluency (B); well-being and sleep quality (C). CPM = Colored Progressive Matrices; DS = Digit Span Test; CBT = Corsi Block-Tapping Test; FPT = Five Points Test; VSWQ = Very Short Well-being Questionnaire; SDSC = Sleep Disturbance Scale for Children.

The participant demonstrated improvement after treatment, as evidenced by higher scores in the CPM test [baseline z-score = 0.43; treatment z-score = 0.78; improvement (Δ) = 0.36], which evaluates general cognitive activity. Additionally, there was an improvement in verbal working memory, assessed by Digit Span Test (DS) [baseline z-score = 0.6; treatment z-score = 2.6; improvement (Δ) = 2]. However, no relevant improvement was observed in spatial working memory, as assessed by the Corsi Block-Tapping Test (CBT). All visual outputs are presented in Figure 3A.

### 3.3. Graphic fluency assessment

In graphic fluency, the participant showed robust progress, specifically in the Production index of the FPT [baseline z-score = 1.78; treatment z-score = 3.33; improvement (Δ) = 1.54], as well as in the rule breaking [baseline z-score = 1.68; treatment z-score = −0.32; improvement (Δ) = 2] and perseverations indices [baseline z-score = 0.17; treatment z-score = −0.66; improvement (Δ) = 0.83]. All visual outputs are presented in Figure 3B.

### 3.4. Well-being and sleep quality assessment

Finally, the participant showed a notable improvement in both well-being (VSWQ) [baseline z-score = −0.83; treatment z-score = −0.13; improvement (Δ) = 0.70] and sleep quality (SDSC) following the treatment intervention [baseline z-score = 2.33; treatment z-score = 1.03; improvement (Δ) = 1.30] (Figure 3C).

**Figure 4.**
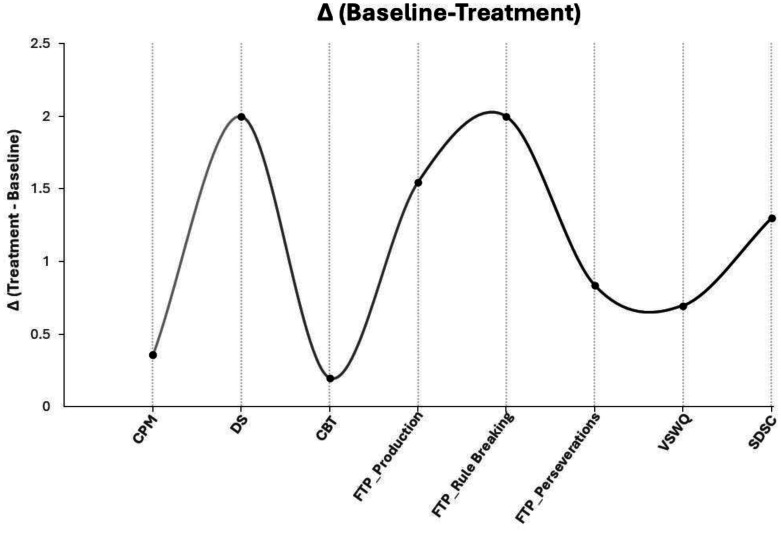
Participant improvements (Δ) in all tests. CPM = Colored Progressive Matrices; DS = Digit Span Test; CBT = Corsi Block-Tapping Test; FPT = Five Points Test; VSWQ = Very Short Well-being Questionnaire; SDSC = Sleep Disturbance Scale for Children.

All participant improvements are shown in Figure 4.

### 3.5. Orthodontic correction

**Figure 5.**
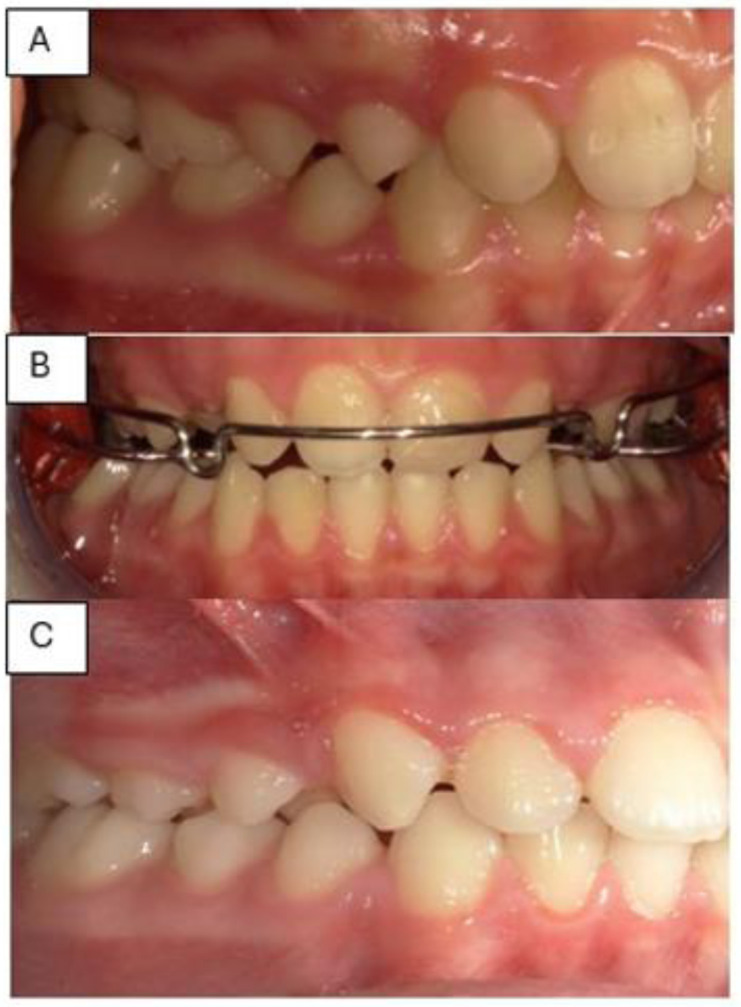
Image of the malocclusion unilateral right posterior crossbite before therapy (A). The appliance FGB in the mouth during treatment (B). Image of the occlusal correction after therapy (C).

The occlusion after functional therapy with FGB was restored, and the buccal cusps of the upper right permanent molar teeth in the frontal plane, which were previously in crossbite, overlapped the lower teeth, thus providing the appropriate physiological stimuli from peripheral receptors and proprioceptors. Also, the molar relationship in the sagittal plane and the anterior incisor guidance improved and were corrected, due to the capability of the functional appliance to work in the three planes of the space (Figure 5).

### 3.6. Chewing pattern recording and kinematic analysis

**Figure 6.**
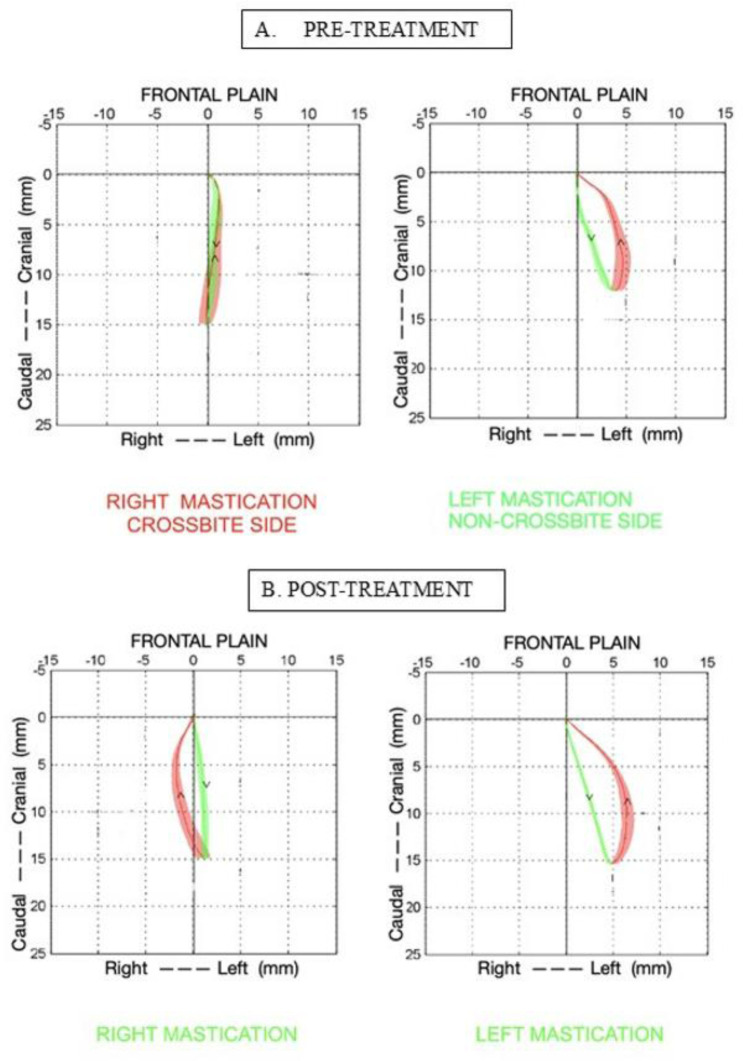
Chewing pattern recording pre-treatment. Masticatory kinematic pattern in the frontal plane of the patient with right posterior unilateral crossbite during chewing of a hard bolus deliberately on the right (crossbite) side shows an anomalous reverse chewing pattern; on the left, it shows a physiological chewing pattern. The solid line, downward arrow for the opening, and upward arrow for the closing pattern represent the average chewing cycle of 3 trials lasting 10 s each; the areas represent the standard deviation over the average cycle (A). Chewing pattern recording post-treatment. The patterns on the right (previous crossbite side) and on the left side (previous normal side) show the correction of the masticatory function (B).

**Figure 7.**
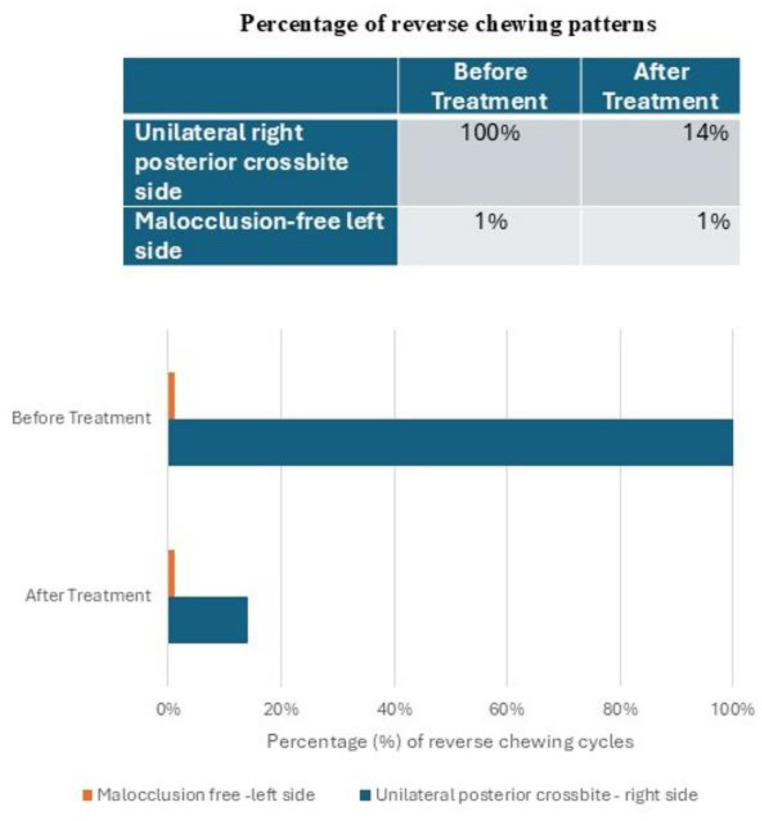
Percentages of altered (reverse) chewing patterns pre- and post-treatment. Unilateral right posterior crossbite side (dark gray) and malocclusion-free left side (light gray). See the Methods section for the calculation of reverse chewing patterns.

Before therapy with the right posterior crossbite, the recording of the chewing function showed a 100% (50/50) of anomalous reverse chewing patterns while chewing a hard bolus deliberately on the right crossbite side. As shown in Figure 7, the reverse chewing patterns develop along the vertical axis, and the opening tracing overlaps the closing, resulting in a narrow pattern with reduced efficiency (Figure 6). After correction of the right unilateral posterior crossbite, the recording of the chewing function showed an important improvement of the chewing patterns, with a significant reduction of the anomalous reverse chewing patterns: from 100% before therapy, it lowered to 14% (7/50) after therapy. While chewing on the right side (the previous crossbite side), the pattern shows a physiological morphology, the opening and closing tracings are no longer overlapped, and the indices of efficiency are restored (Figure 7).

## 4. Discussion

The present single case shows the intrinsic relationship between chewing and cognitive functioning, at the same time highlighting that the functional therapy of a unilateral posterior crossbite with an FGB appliance is able to correct the dental malocclusion and the masticatory function. This is an original study, and to the best of our knowledge, it is being published for the first time.

In particular, a 12-year-old child with a right unilateral posterior crossbite was treated for seven months with an FGB appliance. Both dental, chewing, cognitive, and behavioral assessments were performed before and after treatment to ascertain the baseline level (pre-treatment) and evaluate the results obtained (post-treatment).

From a dental point of view, we can observe that FGC is able to resolve the dental and functional alterations of the unilateral posterior crossbite. In fact, the recording of chewing patterns and the kinematic analysis showed a significant improvement after the treatment period compared to the starting condition. This result is fully in agreement with other clinical observations in which FGB was used [25],[26]. Namely, it has been observed that FGB allows the spontaneous repositioning of the mandible in a centric position with intermittent and self-regulating forces, accomplishing its actions and effects during swallowing and phonation. The recovery of the reverse chewing patterns is the first important step to let the neuromuscular system's memory reprogram itself in a harmonious way [27]. The correction of the reverse chewing pattern is a very important result, as reverse chewing patterns are known to be related to a severe loss of activation and coordination of the masticatory muscle; as soon as the reverse chewing patterns decrease, the muscular balance is restored, and motor control changes [26]. The recovery of the chewing pattern and function took place within 7 months, in agreement with other studies [27].

Although orthodontic aspects are relevant, in this context, we want to emphasize the cognitive and well-being aspects related to chewing. Before starting the treatment, the child observed with malocclusion presented globally normal indices of expression of cognitive activity, which, however, improved significantly after the resolution of the unilateral posterior crossbite. In particular, after the treatment, general cognitive activity measured by means of colored Raven matrices improved, as did verbal short-term memory and graphic fluency. It is important to underline that the choice to specifically investigate short-term memory and graphic fluency abilities is supported by the fact that, in experimental animal studies, chewing impairment was correlated with a visuospatial deficit [1],[8]–[10].

These findings are consistent with previous studies that highlight how impaired chewing is related to a cognitive deficit [28]. For example, animals fed liquid and semi-liquid diets were unable to explore familiar and novel environments efficiently [10]. As previously underlined, a possible explanation of the effects of correct/altered chewing on the expression of cognitive abilities is to be found in the anatomical–functional connections that from the trigeminal nucleus reach the locus coeruleus and from there extend in a widespread manner throughout the neocortex (noradrenergic system) and, in a more specific way, to the hippocampus (dopaminergic system) [29].

Another interesting finding relates to well-being and sleep. The child observed doubled scores on the well-being scale after the treatment, suggesting that correct chewing is also a prerequisite for well- being. The progress on sleep quality is also very interesting. Before starting treatment, our child had a critical score. After the correction of the malocclusion, a significant improvement can also be observed in the quality of sleep. Sleep is an indispensable physiological process. Sleeping well also means best consolidating the information acquired while awake [30]. Also for this reason, sleep is essential for growth. Correcting posterior unilateral crossbite malocclusion together with the restoration of the masticatory function and the related muscular coordination [27],[31], which is the real aim of early therapies with functional appliances (FGB), means offering the child the opportunity of healthy growth and the possibility of developing their cognitive potential.

## 5. Limitations

While this study contributes valuable insights into the potential cognitive benefits associated with the use of an FGB appliance for treating unilateral posterior crossbite, several limitations warrant consideration. First, the study's reliance on a singular case restricts the generalizability of findings to broader populations or contexts. For this reason, the generalizability of the findings is inherently limited due to the individual nature of the study. Single-case designs inherently lack the statistical power and sample size required for robust conclusions or extrapolation beyond the specific case studied. Moreover, the absence of a control group or comparative analysis limits the ability to establish causal relationships between FGB treatment and cognitive outcomes. In fact, without a control group, causal inference is weakened, limiting the capacity to attribute cognitive improvements solely to the orthodontic intervention, weakening the interpretive power of the FGB treatment. Factors such as individual variability or educational factors, environmental influences, developmental maturation, and potential confounding variables are not adequately controlled in single-case studies, raising questions about the reliability and validity of observed effects.

Additionally, while the study assesses cognitive domains like global cognitive activity, verbal and spatial working memory, and graphic fluency, the scope of cognitive assessment may not fully capture all cognitive functions influenced by FGB treatment. Finally, this study does not allow evaluating whether improvements in cognition and well-being depend only on improvements in occlusal status rather than on improvements in sleep quality. However, the evidence showing that correct chewing is strongly correlated with cognitive processes and a better quality of sleep is starting to be consistent [27]–[31]. At the same time, however, to understand the relationship between chewing and cognition, it would be helpful to investigate the relationship between sleep and cognition more accurately, since several studies highlight how good sleep efficiency correlates with better attention [30]. Despite these limitations, this study serves as a valuable starting point for further research into the intricate interplay between masticatory function, orthodontic interventions, and cognitive functioning. Larger-scale studies with rigorous experimental designs are needed to better understand the underlying mechanisms and therapeutic implications in diverse populations and contexts.

## 6. Conclusions

In conclusion, this study provides valuable insights into the potential cognitive benefits associated with addressing unilateral posterior crossbite functional treatment. Despite limitations, the findings underscore the importance of considering physiological and non-traumatic orthodontic interventions in the context of cognitive functioning and psychological well-being. The observed improvements in chewing function and cognitive abilities following treatment with the function generating bite appliance highlight the possible interconnectedness between masticatory function and cognitive performance. However, further research with larger sample sizes and robust experimental designs is needed to validate these original findings and unravel the underlying mechanisms. Such endeavors are essential for advancing integrated healthcare practices that take into account dental care alongside functional, cognitive, and psychological aspects, ultimately promoting comprehensive well-being and optimal development. This study serves as a valuable starting point for future investigations into the complex relationship between orthodontic treatments, mastication, cognitive function, and overall health outcomes.

## Use of AI tools declaration

The authors declare they have not used Artificial Intelligence (AI) tools in the creation of this article.
